# Rapid Detection of Aflatoxins in Ground Maize Using Near Infrared Spectroscopy

**DOI:** 10.3390/toxins16090385

**Published:** 2024-09-04

**Authors:** Sylviane Bailly, Béatrice Orlando, Jean Brustel, Jean-Denis Bailly, Cecile Levasseur-Garcia

**Affiliations:** 1 Mycoscopia, 3 rue Jean Monnet, 31470 Fonsorbes, France; mycoscopia@gmail.com; 2 Arvalis Institut du Végétal, Station Expérimentale, 91720 Boigneville, France; b.orlando@arvalis.fr; 3 Physiologie, Pathologie et Génétique Végétales (PPGV), Université de Toulouse, INP-PURPAN, 75 Voie du Toec, 31076 Toulouse, France; jean.brustel@gmail.com; 4 Laboratoire de Chimie Agro-Industrielle (LCA), Université de Toulouse, INRAE, INPT, École Nationale Vétérinaire de Toulouse, 23 Chemin des Capelles, 31076 Toulouse, France; 5 Laboratoire de Chimie Agro-Industrielle (LCA), Université de Toulouse INRAE INPT, INP-PURPAN, 31076 Toulouse, France

**Keywords:** aflatoxins, maize, rapid detection, near infrared spectroscopy

## Abstract

Aflatoxins are carcinogenic mycotoxins that may contaminate many crops and more especially maize. To protect consumers from these contaminants, many countries set up low regulatory thresholds of few µg/kg. The control of food requires time-consuming analysis for which sampling is a key step. It would therefore of key sanitary and economic relevance to develop rapid, sensitive and accurate methods that could even be applied on line at harvest, to identify batches to be excluded as soon as possible. In this study, we analyzed more than 500 maize samples taken at harvest during 3 years for their aflatoxin contamination using HPLC-MS. Among them, only 7% were contaminated but sometimes at levels largely exceeding European regulations. We demonstrate that Near InfraRed Spectroscopy (NIRS) could be of great help to classify cereal samples according to their level of aflatoxin contamination (below or higher than E.U. regulation). To build the model, all AF contaminated samples as well as an equivalent number of AF free samples were used. NIRS performance was not sufficient to quantify the toxins with adequate precision. However, its ability to discriminate naturally contaminated maize samples according to their level of contamination with aflatoxins in relation to European regulations using a quadratic PCA-DA model was excellent. Accuracy of the model was 97.4% for aflatoxin B1 and 100% for total aflatoxins.

## 1. Introduction

Aflatoxins (AF) are mycotoxins produced by *Aspergillus* section *Flavi* and that may contaminate many crops [1]. Among cereals, maize is the most susceptible crop and it may be contaminated both in the field, before harvest, or later during storage in case of insufficient drying or remoistening [2]. Both aflatoxin B1 (AFB1) and the mixture of AF (B1, B2, G1 and G2) are classified by IARC in the group I of carcinogenic compounds for Human [3]. Indeed, they are genotoxic, mutagenic and responsible for the appearance of hepatocarcinoma [4]. AF were also demonstrated to induce growth retardation in children and to be toxic for the immune system [5,6]. AFB1 is among the natural contaminants leading to the highest number of DALYs (Disabled Adjusted Life Years) [7].

The toxicity of AF led more than 100 countries to set up regulations to prevent consumers’ exposure to these contaminants [8]. As an illustration, for AFB1, in Europe, these limits range between 2 and 12 µg/kg in foods and from 5 to 20 µg/kg in feed as a function of the species [9]. Due to the existence of these strict regulatory limits, the classification of crop batches according to their level of contamination with AF at harvest is of primary importance to be able to sort and orient them towards the best possible usage.

To date, the gold method for AF analysis relies on HPLC with different modes of toxin detection. The natural fluorescence of these molecules being often used to detect them after liquid chromatography separation [10]. However, such method requires expensive equipment and is highly dependent of the quality of the sampling. Indeed, mycotoxin contamination is generally heterogeneous, and results may strongly differ as a function of the sampling methodology. Some rules were set up to define the correct sampling procedure for mycotoxin analysis of food and feed batches [11]. But their realization in practice is often complicated due to the high number of subsamples that shall be taken to be representative of a whole batch. Another important limitation is time required for analysis since, for cereals, the time between harvest and storage is often very limited. It is not possible to block batches waiting for the results of the analysis. Other rapid methods were developed for AF analysis, such as immunoassays [12]. But, as HPLC, most are destructive and results directly depends on the sampling quality.

Within this context, the development of analytical methods that could allow an online, non-destructive analysis of AF in grains at harvest would be of major interest. It could help sort grains as a function of their level of contamination and direct them towards the best valorization possible. Among possible methodologies, Near InfraRed Spectroscopy (NIRS) emerge as a good candidate since rapid, non-destructive, and able to simultaneously measure multiple components in complex matrices [13].

NIRS uses Near InfraRed light (800–2500 nm) to analyze the chemical properties of samples. By absorbing specific wavelengths, chemical bonds (C, H, O, N) reveal composition and molecular structure. Applied in agriculture, feed and food industry, pharmaceuticals, environmental monitoring, NIRS is fast, non-destructive, and requires no complex sample preparation.

Many studies have used NIRS to quantify diverse mycotoxins in food and feed [for review, see [14]]. Regarding AFB1 detection, some recent works tried to use NIR spectroscopy to analyze maize grains. Wang (2022) proposed a novel approach for monitoring AFB1 content in maize using deep learning models based on NIR spectra, with the following performance: a Root Mean Square Error of Prediction (RMSEP) of 1.3591 µg/kg and a predictive determination coefficient of 0.9955 [15]. Deng (2022) created Support Vector Machine (SVM) models with an RMSEP of 3.5967 µg/kg, a determination coefficient (R^2^) of 0.9707, and a Relative Prediction Deviation (RPD) of 5.7538 [16]. Finally, Liu (2022) established back propagation neural network (BPNN) models with a correlation coefficient of 0.9951 and a RMSEP of 1.5606 µg/kg [17]. The main conclusions were that quantification of mycotoxins is possible but not precise enough for targeted thresholds, notably for AFB1 for which very low regulatory thresholds of few µg/kg are set for food in Europe [9]. Moreover, these studies were done using artificially contaminated maize, which can modify spectral response compared to naturally contaminated one.

Within this context, the objective of this study was to develop a new approach based on NIR spectroscopy aiming not to quantify AFB1 in samples but to sort them according to their risk of contamination above or under the regulatory limit of 2 µg/kg for AFB1 and/or 4 µg/kg for total AF, using a set of naturally contaminated samples harvested during three years in French fields.

To develop a method that can be further transferred to the maize sector, we collected spectra using a portable spectrometer which offers numerous advantages: it is compact and easy to carry, allowing for on-site analysis and real-time data collection in various locations (as in silos for grain storage). It can be used directly in real industrial environments, providing accurate and immediate measurements without the need for sample transportation to a laboratory. Additionally, portable spectrometers are generally less expensive compared to traditional laboratory spectrometers, making them a cost-effective solution. These devices are designed to be user-friendly, requiring minimal training to operate, which enhances efficiency and reduces the likelihood of errors. The simplicity and affordability of portable spectrometers also make advanced analytical techniques accessible to a broader range of users, including small businesses and educational institutions.

## 2. Results and Discussion

### 2.1. Sample Contamination with Aflatoxins

A total of 554 samples was collected from French fields at harvest during 2018–2020. They were analyzed for aflatoxin contamination using HPLC-MS. Table 1 summarizes the obtained results.

Aflatoxins are still emerging contaminants in France and more than 90% of the samples had no detectable levels of aflatoxins, indicating a large number of samples were either free of aflatoxins or contained them below detectable levels. The maximum level of AFB1 detected was 30.17 μg/kg, while the total AF reached up to 73.07 μg/kg. The mean levels of contamination in contaminated samples were 3.78 µg/kg and 5.94 µg/kg for AFB1 and the sum of AF, respectively. It decreased to 0.270 μg/kg and 0.435 μg/kg when considering all samples.

Aflatoxin B1 showed a variance of 55.58 μg/kg and a standard deviation of 7.46 μg/kg, indicating high variability in the data. Total AF had a higher variance of 183.09 μg/kg and a standard deviation of 13.53 μg/kg, showing greater dispersion and a few extreme values influencing the dataset.

The European Union (EU) has set strict regulatory limits for AF in food to protect public health [9]. Only 2% (n = 11) of samples exceeded the limit of 2 µg AFB1/kg and 1.6% (n = 9) exceeded the limit for total AF content set at 4 µg/kg. Even if in low proportion, contaminated samples sometimes displayed quite high levels of contamination, highlighting the need to identify and exclude them from Human consumption as soon as possible at harvest, and before storing them. Indeed, AF contamination can increase during storage in case of development of aflatoxigenic species due to incomplete drying or remoistening [18]. Such detection requires a rapid method, possibly usable online.

The aim of this study was to develop an infrared prediction model for aflatoxin levels. However, the predominance of samples with low contamination levels could bias the model. To mitigate this, we conducted a targeted sampling that included all samples with detectable aflatoxin levels and an equal number of samples with non-detectable levels. Considering the three years of data, 2018, 2019, and 2020, a Kennard and Stone sampling was performed to select 38 samples with undetectable total aflatoxin levels. These samples were issued from 2018 (n = 7), 2019 (n = 4) and 2020 (n = 28) harvest to mimic the number of contaminated samples for these three years.

### 2.2. Quantification of Aflatoxins Contents Using NIRS

#### 2.2.1. Spectra Acquisition

**Figure 1:**
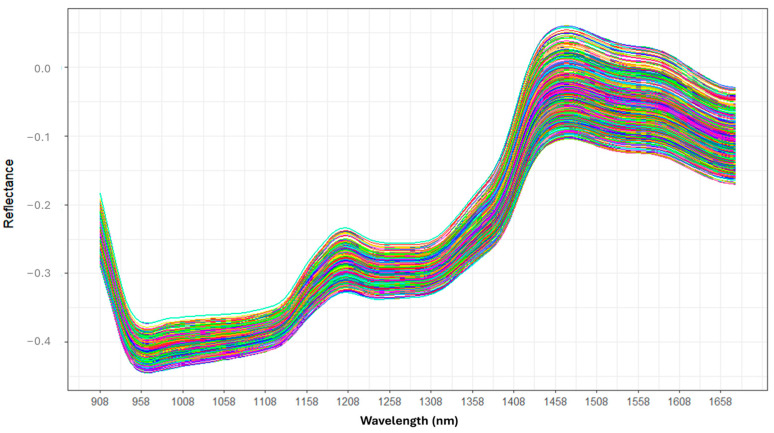
Raw spectra of maize samples across Wavelengths 908–1650 nm.

A MicroNIR OnSite spectrometer (Viavi Solutions Inc., San Jose, CA, USA) was used in reflectance mode to acquire NIR spectra from crushed grains over the spectral range of 908–1676 nm, consisting of 125 variables. Each spectrum represents the average of 100 scans, taken with an integration time of 10 ms and a spectral resolution of 6 nm. The raw spectra of all samples are presented on Figure 1.

The spectra show notable variations in reflectance, especially in the 900–1000 nm region, which shows a rapid decrease with a significant dip around 960 nm, possibly due to absorptions from water (OH) and organic compounds. The 1100–1300 nm zone is stable with slightly increasing reflectance. In the 1400–1500 nm range, there is a notable increase, plateauing around 1460 nm, often due to the first harmonic of OH and NH bonds. After 1500 nm, reflectance slightly decreases. These spectral variations may indicate differences in the composition and properties of the samples, likely due to the presence of fungi, their metabolism, and the resulting modifications in the maize [19].

#### 2.2.2. Partial Least Squares Regression (PLS) Model

A PLS model was developed using AFB1 and total AF contents as the response variables and wavelengths (125 variables) as the explanatory variables. This approach effectively handles collinear and noisy spectral data, reduces dimensionality, and preserves relevant information, resulting in more accurate predictions of AF levels and better contamination monitoring. The model was developed using 80% of the samples, selected with the Kennard and Stone algorithm, and validated on the remaining 20%. So, the calibration set included 54 samples, while the validation set included 12 samples. The percentage of contaminated samples (AFB1 content > 2 µg/kg) was 50% in both the calibration and validation sets. Several commonly used mathematical preprocessings in NIRS were tested on the raw spectra. The modeling on SNV (Standard Normal Variate) + D1 (first derivative) preprocessed spectra showed the best performance for predicting AFB1 content in ground corn samples with a determination coefficient r^2^cv = 0.97, a Root Mean Square Error of Cross-Validation (RMSECV) of 1.44 µg/kg, a Ratio of Performance to Deviation in Cross-Validation (RPDcv) of 6.3, a r^2^test of 0.56, a Root Mean Square Error of Prediction (RMSEP) of 6.6 µg/kg, and a RPDtest of 1.4. The best PLS regression model performance was also obtained on SNV + D1 preprocessed spectra for total AF: r^2^cv = 0.99, RMSECV = 1.88 µg/kg, RPDcv = 9.0, r^2^test = 0.78, RMSEP = 8.10 µg/kg, and RPDtest = 2.1 (Table 2).

SNV + D1 preprocessing can enhance the spectral data by removing multiplicative scatter effects and baseline shifts, leading to more accurate and reliable predictions [20].

The use of SNV+D1 preprocessing improved the model’s performance, highlighting the importance of data preparation for spectroscopy models [21]. There is a noticeable difference between the cross-validation and test set performances, especially for AFB1, as for Darnell et al. [22]. This may indicate that the model is too specific to the calibration data (overfitting). However, the performances are interesting, as found by other authors [16,21,23]. But the RMSE values were still too high for targeted thresholds. To mitigate this issue, increasing the number of calibration samples or using more robust regularization techniques could be beneficial.

The model appeared to perform better in predicting total AF compared to AFB1. This could be due to clearer spectroscopic signals associated with maize modifications due to AF and fungi presence.

In conclusion, although the calibration results were excellent, the test set performance indicated that there is still room for improvement to meet the European regulatory thresholds.

#### 2.2.3. Artificial Neural Network (ANN) Model Regression

To address the limitations of PLS models, ANN models were developed to predict aflatoxins contents, tuning hyperparameters to optimize the model. Neural networks, with their ability to model complex, non-linear relationships, might offer better precision and generalization compared to traditional PLS regression [24].

**Figure 2:**
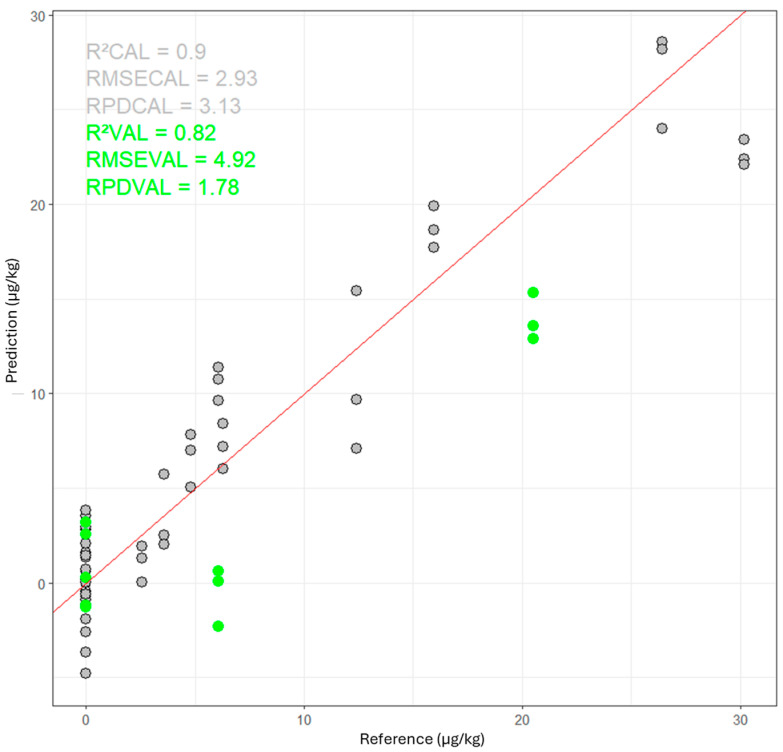
Performance of the ANN model for predicting AFB1 content in ground maize samples. The scatter plot shows the predicted versus reference values for the training set (grey circles) and the validation set (green circles).

The best ANN model for predicting AFB1 was developed using SNV+D1 preprocessed spectra. It had the following characteristics: a hidden layer with 72 neurons, sine activation function, and initial weight assignment randomly following a negative uniform distribution (from −1 to 1). The performance for predicting the AFB1 content in maize samples was: r^2^cv = 0.90, RMSECV = 2.93 µg/kg, RPDcv = 3.1, r^2^test = 0.82, RMSEP = 4.9 µg/kg et RPDtest = 1.8 (Figure 2).

So, the ANN model for AFB1 showed good performance in calibration. However, the performance on the validation set was slightly lower, indicating overfitting of the calibration and some difficulty in generalizing the model to new data. This could be due to the complexity of sample variability or subtle differences in the spectra not captured by the calibration model. The size of the calibration set is limited for ANN training, which displays its full potential from 500 to 1000 calibration samples. Enriching the data set, particularly with spectra from contaminated samples, would help the models to better address their specificities and deliver better performance, while avoiding overlearning.

**Figure 3:**
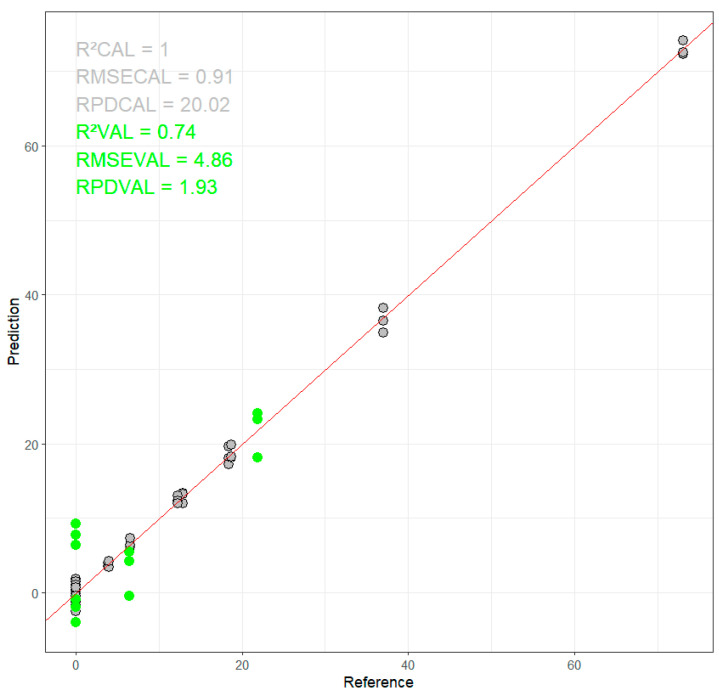
Performance of the ANN model for predicting Total AF content in maize samples. The scatter plot shows the predicted versus reference values for the training set (grey circles) and the validation set (green circles).

The best ANN model for predicting total AF content has the following characteristics: 33 neurons in the hidden layer, sine activation function, and initial weight assignment randomly following a negative uniform distribution (from −1 to 1). The modeling on SNV + D1 pretreated spectra was the best for predicting total aflatoxin content in maize samples: r^2^cv = 0.99, RMSECV = 0.91 µg/kg, RPDcv = 20.0, r^2^test = 0.74, RMSEP = 4.9 µg/kg et RPDtest = 1.9 (Figure 3).

The ANN model for total AF showed excellent performance in calibration, indicating the model’s ability to explain almost all the variance in the training data. The performance on the test set was also good, although slightly lower than that of the training set, which was expected. This shows that the model is relatively robust but could still benefit from more data or techniques to improve generalization.

The performance of the best ANN models is recapitulated in Table 3.

To further optimize, we could test other types of algorithms, such as extreme gradient boosting (XGBoost), which is an advanced implementation of gradient-boosted decision trees designed for speed and performance [25]. Additionally, to improve generalization, it would be beneficial to add more samples, include data from more harvest years, and consider testing a round-robin method on the harvest years. The round-robin method involves using data from different years in a rotating mode to ensure that the model generalizes well across different time periods [26].

Overall, the ANN models showed significant improvement compared to PLS models, especially in terms of r^2^ and RMSE, as already demonstrated by other studies [15,17]. This suggests that ANNs can capture complex nonlinear relationships in the data, that PLS models cannot do as effectively. However, improving performance on test sets remains a challenge, highlighting the need for representative sampling and rigorous validation to ensure the models generalize well.

Although the ANN models showed promising performance, their practical use for predicting aflatoxin content must be carefully validated, particularly to ensure they meet regulatory thresholds. Therefore, a classification approach for maize samples was then tested.

### 2.3. Evaluation of the Aflatoxin Risk by NIRS

#### Characteristics and Performances of the Best Prediction Models for Aflatoxin Content Classes from NIR Spectra

Five mathematical preprocessings—raw, D1, D2, D3, and SNVD—were tested on the maize samples’ spectra. The raw preprocessing uses the data without modification. D1, D2, and D3 correspond to the first, second, and third derivatives of the spectra, respectively, to enhance specific features and reduce baseline variations. SNVD (Standard Normal Variate De-trending) normalizes the spectra to correct for scatter and offset variations. These preprocessings improve the quality of spectral data, enabling more accurate analysis of aflatoxins contents. The variables to explain were AFB1 class (less or more than 2 µg/kg) and total AF (less or more than 4 µg/kg). The model used was Principal Component Analysis—Discriminant Analysis (PCA-DA). Models were developed with three discriminant algorithms: Linear Discriminant Analysis (LDA), Quadratic Discriminant Analysis (QDA), and Mahalanobis Distance-based Discriminant Analysis (MDA). The quadratic model on SNVD yielded the best cross-validation performance for both AFB1 and total AF.

The quadratic PCA-DA model was preferred over linear PCA-DA because aflatoxins content classes are likely not linearly separable in the variable space after PCA dimension reduction. Quadratic discriminant analysis can model non-linear decision boundaries, thereby enhancing its ability to capture differences between classes [27].

The model’s performances were based on AFB1 contamination of maize, with the following categories: “lower2” (usable maize, containing less than 2 µg AFB1/kg) and “higher2” (maize to be exclude from Human consumption, containing more than 2 µg AFB1/kg). The presented performances were obtained through cross-validation.

In the context of the confusion matrix provided, positive cases were the samples that were contaminated (with more than 2 µg/kg of AFB1). Negative cases were the samples that were not contaminated (AFB1 lower than 2 µg/kg). The acceptance of batches with contaminated corn kernels (false negatives) poses a food safety risk for consumers, while the rejection of batches with mostly harmless corn kernels (false positives) represents an economic loss for producers.

The confusion matrix revealed the following: there were 11 true positives (correctly identified contaminated maize samples), 63 true negatives (correctly identified non-contaminated maize samples), 2 false positives (non-contaminated maize samples incorrectly identified as contaminated), and 0 false negatives (non-identified contaminated maize samples).

For AFB1, the summary is as follows: accuracy (the proportion of true results among the total number of cases examined): 97.4%, precision (the proportion of true positive results among all positive results predicted by the model): 84.6%, recall (sensitivity; the proportion of true positive results among all actual positive cases): 100%, and F1 score (the harmonic mean of precision and recall): 91.7%. These metrics provide a comprehensive evaluation of the model’s performance in cross-validation. The results showed very high accuracy and perfect sensitivity, which is essential to ensure that all contaminated maize is correctly identified and excluded from Human consumption. Additionally, the high precision and F1 score indicate that the model made few errors in identifying non-contaminated maize as contaminated (false positives), while maintaining good overall performance.

For total AF, the performance metrics were as follows: accuracy: 100%, precision: 100%, recall (Sensitivity): 100%, and F1 score: 100%. These performance metrics indicate that the model’s predictions for total AF contamination in maize were perfect. The accuracy of 100% means that all predictions made by the model were correct. The precision of 100% shows that every sample predicted as contaminated were indeed contaminated, while the recall of 100% demonstrates that the model successfully identified all contaminated samples. This performance is ideal in ensuring compliance with safety regulations, as it guarantees that all ground contaminated maize is correctly identified and also avoid economic losses since no uncontaminated maize is incorrectly classified.

The performance of our models is summarized in Table 4.

The performance was comparable to those reported in other studies [28], even though several of these studies used significantly higher thresholds [28,29,30]. Additionally, our models outperformed others conducted with larger sample sizes [22,31]. However, it is important to note that these results were obtained through cross-validation on only 76 ground samples. Indeed, in this study, a balanced class distribution was maintained to mitigate potential biases during model training. However, it is important to note that this approach does not entirely represent real-world conditions, where aflatoxin contamination in commercial maize is relatively rare, even if increasing due to climate changes. As such, future research could benefit from exploring how the model performs with an increased test size, incorporating all available samples to better reflect the actual distribution of contamination. So, while the performance is very encouraging, it should be interpreted with caution and require further validation steps.

SNVD preprocessing of spectra was identified as the most effective one, as in the study by Ferandez-Ibanez et al. [21]. This choice was made based on the classification performance. SNVD reduced interferences from noise and background variations in NIR spectra. SNVD amplifies weak signals, facilitating the detection of compounds of interest.

## 3. Conclusions

AF are major contaminants in many hot regions of the world and emerging threats in Europe due to climate changes. These mycotoxins are potent carcinogenic compounds in Human, justifying the set of very low regulatory limits in food but also feed due to the possible carry over of toxic metabolites in milk. The development of rapid detection methods is of key importance, especially in the maize sector since maize is one of the most susceptible crops to AF contamination. In our study, a Partial Least Square (PLS) model developed using Standard Normal Variate + First derivative (SNV + D1) preprocessing achieved good performance with r^2^ = 0.78, and RMSEP = 8.10 µg/kg for quantifying total AF, and r^2^ = 0.56, and a Root Mean Square Error of Cross-Validation = 6.6 µg/kg for AFB1 content. Artificial Neural Network (ANN) models showed significant improvement over PLS models (RMSEP = 4.9 µg/kg for both Total AF and AFB1), yet still lacked precision compared to European regulatory limits. Principal Component Analysis and Discriminant Analysis (PCA-DA) was then used to classify ground maize samples based on European regulatory limits, achieving high performance metrics: accuracy = 97.4% for AFB1 and 100% for total AF. Our study thus demonstrated that NIR spectroscopy could be of great interest to classify ground maize samples according to their level of contamination. The use of Quadratic PCA-DA models allowed accurate, precise and sensitive classification of samples according to their compliance with EU regulatory limits for AFB1 and/or total AF. Inclusion of other samples and test on whole grains are now required to better validate these models and evaluate their transferability to the maize sector.

## 4. Materials and Methods

### 4.1. Maize Samples

A total of 554 samples was collected in French maize fields during 2018–2020 period (195 in 2018; 183 in 2019 and 176 in 2020). In each field, sampling was done at harvest by taking 3 times 1.5 kg of grains during combine harvester emptying, at three different periods. The three subsamples were mixed to obtain a 4.5 final sample. They were dried 24–48 h at 40 °C and cleaned to eliminate impurities. 1.5 kg from each sample were taken and ground for analysis using a hammer mill equipped with a 1 mm sieve.

### 4.2. Aflatoxin Analysis

Aflatoxins B1, B2, G1 and G2 were analyzed in a COFRAC accredited laboratory using liquid chromatography—tandem mass spectrometry as already described [32]. Limits of detection were 0.1, 0.1, 0.125, and 0.25 µg/kg for AFB1, AFB2, AFG1 and AFG2 respectively. Corresponding limits of quantification were 0.25, 0.25, 0.25 and 0.5 µg/kg for AFB1, AFB2, AFG1 and AFG2 respectively.

### 4.3. Collection of NIR Spectra

A MicroNIR OnSite spectrometer (Viavi Solutions Inc., San Jose, CA, USA) was used in reflectance mode to acquire NIR spectra from crushed grains over the spectral range of 908–1676 nm, consisting of 125 variables. Spectra were taken from a small cup containing 5 g of maize sample (FOSS, Hilleroed, Denmark). Each spectrum represents the average of 100 scans, taken with an integration time of 10 ms and a spectral resolution of 6 nm. The averaged reflectance spectra were then converted into absorbance spectra using the Viavi spectrometer software, MicroNIR TM Pro v2.5. Additionally, three spectra were collected for each sample, involving refilling between measurements to ensure consistency and accuracy of the data collected.

### 4.4. Statistical Approaches and Data Mining

The aflatoxin B1 (AFB1) and total aflatoxin (TOT AF) concentrations were discretized to align with the European Union regulations. Specifically, the AFB1 levels were categorized based on whether they were above or below the threshold of 2 µg/kg, while the total AF content was categorized using a threshold of 4 µg/kg. To re-balance the classes of aflatoxin concentrations within the dataset, a stratified random sampling was performed XLSTAT Premium statistical software (version 2023.2.0).

Several preprocessing steps were applied to the spectral data: raw spectra, first, second, and third derivatives using Savitzky-Golay, followed by Standard Normal Variate (SNV) correction combined with detrending and Multiplicative Scatter Correction (MSC). Preprocessing was performed on the spectra before and after averaging the repetitions.

A NIPALS Principal Component Analysis (PCA) was performed to reduce the dimensionality of the spectral dataset. Following this, an Analysis of Variance (ANOVA) was conducted between the principal components (PCs) and the categories of AFB1, distinguishing samples with levels higher or lower than 2 µg/kg. An ANOVA was conducted between the principal components and the AF classes (higher or lower than the threshold), followed by a Tukey post-hoc comparison of group means test. They were done using XLSTAT Premium statistical software (version 2023.2.0).

A Partial Least Squares (PLS) regression was performed, using aflatoxin content as the dependent variable to be predicted and the raw or pre-processed spectra of maize samples as the explanatory variables. The model was developed using 80% of the samples, selected with the Kennard and Stone algorithm (package *prospectr* on R version 4.2.2). The model was validated on the remaining 20%. PLS was developed using the mdatools package on R version 4.2.2.

Artificial Neural Network (ANN) models were developed with the elmNNRcpp package, implemented in R version 4.2.2. Hyperparameter tuning was performed to optimize the model, adjusting parameters such as the number of neurons per layer, the activation functions, and the initial weight distributions. The activation functions available in the elmNNRcpp package are sigmoid, sine, radial basis, hard-limit, symmetric hard-limit, satlins, tan-sigmoid, triangular basis, rectifier linear unit and linear. Weights can be initially assigned according to a normal distribution, a negative uniform distribution between −1 and 1, or a positive uniform distribution between 0 and 1. All combinations of activation functions and weights distribution were tested.

The performance of PLS and ANN regression models were assessed using the r^2^, RMSE, and RPD metrics, both on the training and testing sets. The coefficient of determination, r^2^, measures the proportion of the total variance of the dependent variable explained by the model. RMSE (Root Mean Squared Error) quantifies the standard deviation of the residuals (prediction errors), providing a measure of the overall accuracy of the model. Finally, RPD (Ratio of Performance to Deviation) compares the precision of the model to the variability in the data, indicating the quality of the prediction relative to the observed variation in the data. The choice of the best model is based on performances on the validation set, with the highest R^2^ and RPD and the lowest RMSE. Then, the Akaike criterion is as low as possible among the best models.

A Linear Discriminant Analysis (LDA) was performed on the spectral data. Discriminant analysis is a supervised classification method. However, because the number of variables exceeded the number of individuals, it was necessary to reduce the dimensionality before applying the LDA algorithm. To achieve this, a Principal Component Analysis (PCA) was done to extract the most significant principal components. This combined approach, known as PCA-DA, addresses the issue of high dimensionality while retaining the most important information from the original data. Three discriminant algorithms were tested, including Linear Discriminant Analysis (LDA), Quadratic Discriminant Analysis (QDA), and Mahalanobis Distance-based Discriminant Analysis, to determine which one provided the best performance on our data. With only 76 samples available, external validation was not performed. Instead, cross-validation was used to evaluate the models. The performance is summarized in a confusion matrix. Several performance metrics were calculated from the confusion matrix. Accuracy is the proportion of correct results (both true positives and true negatives) among all cases examined.

$$Accuracy=\frac{\left(TP+TN\right)}{\left(TP+TN+FP+FN\right)}$$

Precision is the proportion of positive predictions that were correct.

$$Precision=\frac{TP}{\left(TP+FP\right)}$$

Recall (or Sensitivity) is the proportion of actual positives that were correctly identified.

$$Recall=\frac{TP}{\left(TP+FN\right)}$$

F1 Score is the harmonic mean of precision and recall.

$$F1\;Score=\frac{2\times \left(Precision\times Recall\right)}{\left(Precision+Recall\right)}$$

The preprocessing of the spectral data, and the development of the PLS-DA models were done using The Unscrambler (version X, CAMO Software AS, Oslo, Norway).

## Figures and Tables

**Table 1:** Characteristics of AFB1 and Total aflatoxin contamination of the samples included in the study.

	AFB1	Total Aflatoxins
Number of analyzed samples	554	554
Number of contaminated samples	38	39
Minimum (μg/kg)	0.015	0.015
Maximum (μg/kg)	30.17	73.07
Mean of contaminated samples (μg/kg)	3.78	5.94
Samples > regulation *	11	9
Variance (n − 1) (μg/kg)	55.58	183.09
Standard deviation (n − 1) (μg/kg)	7.46	13.53

* 2 µg/kg for AFB1 alone or 4 µg/kg for the sum of aflatoxins.

**Table 2:** Performances of best NIR models based using partial least squares (PLS) regression for determining AFB1 and total aflatoxins content.

Dataset	n *	Preprocessing	r^2^	RMSE (µg/kg)	RPD
AFB1 calibration	54	SNV + D1	0.97	1.44	6.3
AFB1 testing	12	SNV + D1	0.56	6.6	1.4
Total Aflatoxins calibration	54	SNV + D1	0.99	1.88	9.0
Total Aflatoxins testing	12	SNV + D1	0.78	8.10	2.1

*: number of samples used.

**Table 3:** Performance of the best ANN models for predicting AFB1 and total AF contents in maize samples.

Dataset		Nb. Neurons in the Hidden Layer	Hidden Layer Activation Function	Initial Weight Assignment	Preprocessing	r^2^	RMSE (µg/kg)	RPD
AFB1	Cross validation	72	sine	randomly following a negative uniform distribution (from −1 to 1)	SNV + D1	0.90	2.93	3.1
	Test					0.82	4.9	1.8
Total AF	Cross validation	33	sine	randomly following a negative uniform distribution (from −1 to 1)	SND + D1	0.99	0.91	20.0
	Test					0.74	4.9	1.9

**Table 4:** Performance of the best PCA-DA models for predicting AFB1 and total aflatoxins contents in maize samples (cross-validation on 76 samples).

Dataset	Preprocessing	Accuracy	Precision	Recall	F1-Score
AFB1	SVN + Detrend	97.4%	84.6%	100%	91.7%
Total AF	SVN + Detrend	100%	100%	100%	100%

## Data Availability

Data will be available on request to corresponding author.
